# Using pyrosequencing to shed light on deep mine microbial ecology

**DOI:** 10.1186/1471-2164-7-57

**Published:** 2006-03-20

**Authors:** Robert A Edwards, Beltran Rodriguez-Brito, Linda Wegley, Matthew Haynes, Mya Breitbart, Dean M Peterson, Martin O Saar, Scott Alexander, E Calvin Alexander, Forest Rohwer

**Affiliations:** 1 Department of Biology, San Diego State University, San Diego, USA.; 2 Center for Microbial Sciences, San Diego State University, San Diego, USA.; 3 Computational Science Research Center, San Diego State University, San Diego, USA.; 4 Fellowship for Interpretation of Genomes, Burr Ridge, USA.; 5 Natural Resources Research Institute, Department of Geological Sciences, University of Minnesota, Duluth, USA.; 6 Department of Geology and Geophysics, University of Minnesota, Minneapolis, USA.

## Abstract

**Background:**

Contrasting biological, chemical and hydrogeological analyses highlights the fundamental processes that shape different environments. Generating and interpreting the biological sequence data was a costly and time-consuming process in defining an environment. Here we have used pyrosequencing, a rapid and relatively inexpensive sequencing technology, to generate environmental genome sequences from two sites in the Soudan Mine, Minnesota, USA. These sites were adjacent to each other, but differed significantly in chemistry and hydrogeology.

**Results:**

Comparisons of the microbes and the subsystems identified in the two samples highlighted important differences in metabolic potential in each environment. The microbes were performing distinct biochemistry on the available substrates, and subsystems such as carbon utilization, iron acquisition mechanisms, nitrogen assimilation, and respiratory pathways separated the two communities. Although the correlation between much of the microbial metabolism occurring and the geochemical conditions from which the samples were isolated could be explained, the reason for the presence of many pathways in these environments remains to be determined. Despite being physically close, these two communities were markedly different from each other. In addition, the communities were also completely different from other microbial communities sequenced to date.

**Conclusion:**

We anticipate that pyrosequencing will be widely used to sequence environmental samples because of the speed, cost, and technical advantages. Furthermore, subsystem comparisons rapidly identify the important metabolisms employed by the microbes in different environments.

## Background

Banded iron formations started appearing ~3,700 million years ago when localized sea floor cyanobacterial photosynthesis raised oxygen concentrations high enough that dissolved iron precipitated. That iron powered the industrial revolution. The Soudan Iron Mine in Minnesota, USA was active from 1884 to 1962, and during this period 17.9 million tons of iron ore, primarily hematite, were removed. Nowadays the mine is used as a state park and as a facility for high-energy physics experiments.

Metagenomics is a term used to describe "the functional and sequence-based analysis of the collective microbial genomes contained in an environmental sample"[1,2]. Random shotgun sequencing of DNA from natural communities has been used to characterize seawater, sediment, and fecal viral communities [2-5], as well as the microbial communities in soil, whale falls, seawater and the Iron Mountain acid mine drainage (AMD) [6-10]. Comparative metagenomics was introduced recently[6], identifying those sets of genes that distinguish environmental samples. For example, samples from the surface of the ocean contain significantly more photosynthetic genes than soil or other samples[6,8,10]. We have used comparative metagenomics to characterize the metabolic potential of different environments, and identify those genes, pathways, and subsystems that are more common in any particular environment [11].

Most current sequencing is a modification of the classical Sanger method, where extending DNA fragments are stopped by the random incorporation of a fluorescently labeled ddNTP. The different-sized fragments are then separated using capillary gel electrophoresis and detected with a LASER. Pyrosequencing is a fundamentally different methodology because only one dNTP is added into the reaction at a time [12-14]. If there is a complementary base, then the DNA polymerase catalyzes the reaction and releases pyrophosphate. ATP sulfurylase uses the pyrophosphate to produce ATP in the presence of adenosine 5' phosphosulfate (APS). A Charge-Coupled Device (CCD) measures the light produced when the ATP is used by luciferase to convert luciferin to oxyluciferin. 454 Life Sciences has scaled this process up to be massively parallel, determining the composition of more than 300,000 sequences at once, for approximately the same price as 96 to 192 sequencing reactions performed using traditional chemistries[12]. In addition to the massive parallelization, the 454 technology does not require cloning of the environmental samples, thus eliminating many of the problems that are associated with this step of metagenomics[2].

This report describes the first application of pyrosequencing to environmental samples. From this sequence data, we identify the 16S rDNA sequences present in the sample, and apply new annotation methods to this data using the SEED database[15]. This paper also describes a comprehensive statistical treatment of the genes identified in each sample using a completely novel methodology that exploits the differences between metagenome sequences. We demonstrate that completely unique microbial communities inhabit proximate environments joined by a common watercourse, and that using metagenomics we can identify the unique metabolic potentials prevalent in each environment such as their mechanisms of iron acquisition and respiration. The integration of pyrosequencing, subsystems analysis, comparative metagenomics, statistics, hydrogeology, and chemistry provides a comprehensive systems analysis of the Soudan Mine.

## Results and discussion

### Description of the environmental samples

**Figure 1:**
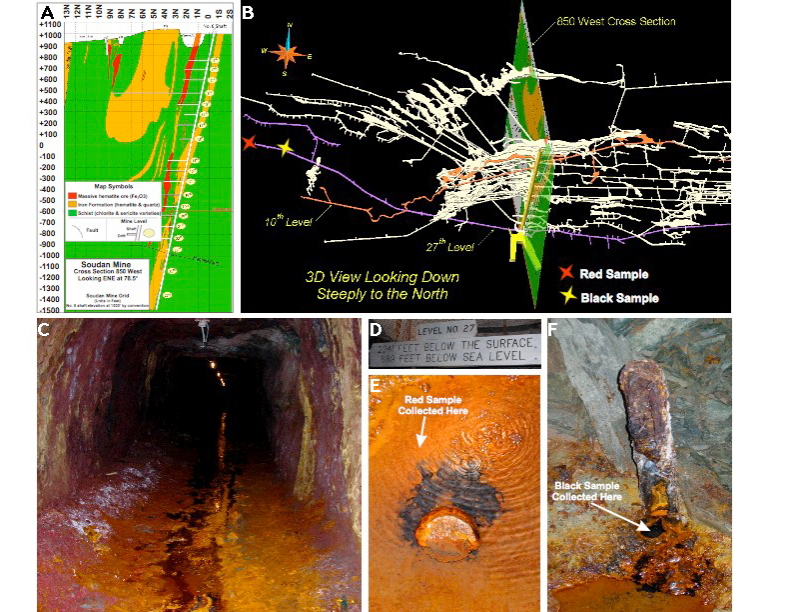
**Sampling from the Soudan Mine**. The Soudan Mine is an Algoma-type Iron Formation rich in hematite. Panel A shows a cross-section of the mine looking East-North-East at 78.5°. Panel B depicts a three dimensional view of the mine, including the cross-section shown in Panel A, and with the sampling sites shown for the "Red" and "Black" samples. Panel C shows the overall flow of water in the mine at level 27, located 714 meters below the surface (Panel D). Panels E and F show a close up of the two sampling sites.

The two environments sampled within the Soudan Mine are shown in Figure 1. Groundwater is not very abundant in the banded iron formations of the mine at our sampling depth of 714 m below the surface. However, small amounts of water emerge steadily from exploration boreholes that extend downward from the deepest level of the mine. The two sets of samples were collected from water trickling out of two such boreholes that are separated by about 100 meters (Figure 1). This water is a calcium, sodium, chloride solution about twice as salty as seawater. It is anoxic, with up to 150 ppm of dissolved ferrous iron and variable enrichments of several trace elements. At both locations, the water emerging from the boreholes produces cm-scale "Black" environments that appear to extend down into the borehole. The water flowing away from each borehole, on the floor of the mine tunnel, is exposed to the oxygenated mine atmosphere, and transitions to a sequence of "Red" environments within a few cm of the orifices. The oxidized environments are continuously fed by anoxic water flowing from the boreholes. The water in the borehole, which yielded the Black sample, as well as a number of similar sites found throughout the mine, has a pH of 6.70 and redox potential of -142 mV. Some of the Black areas are associated with bubbling of gas. The Black sediment contained 5.8 × 10^5 ^microbes per ml. X-ray diffraction analyses of the minerals in this area show that chlorite-serpentine [(Mg,Al)_6_(Si,Al)_4_O_10_(OH)_8_], clinochlore, ferroan [(Mg,Fe)_6_(Si,Al)_4_O_10_(OH)_8_], quartz, and silinaite [LiNaSiO_5_·HCl] are present in the Black sediments. Water slowly flows from the borehole into the stream running down the main mine tunnel. As the water comes in contact with oxygen in the passageway, the pH rapidly decreases to 4.37 and redox potential increases to -8 mV. The Red sample contained 1.2 × 10^6 ^microbes per ml, and these sediments include goethite [FeO(OH)], followed by szaibelyite [(Mg,Mn)BO_2_(OH)], and sussexite [(Mn,Mg)BO_2_(OH)].

### The first two pyrosequences of environmental samples

DNA was purified from the two samples, amplified using the GenomiPhi procedure (GE Healthcare, Piscataway, NJ), and then sequenced by 454 Life Sciences. A summary of the sequence characteristics determined using the pyrosequencing technique is shown in Table 1. The raw sequence reads and quality scores [see Additional files 6 and 7] are provided in compressed format.

**Table 1:** Summary of pyrosequence data from the Soudan Mine

	***Red Sample***	***Black Sample***
Number of Sequences	334,386	388,627
Total Length of Sequences	35,439,683 bp	38,502,057 bp
Average Length of Sequences	106.0 bp	99.1 bp
Average Quality Score^1^	26.2	25.8
Skew^2^	2.53	2.44

^1 ^The quality score of each base was provided by 454 Life Sciences, and is analogous to the Phred score of Sanger Sequencing methods [26]. The value cited here is the average of the mean quality score per sequence.

^2^The skew was calculated by comparing the dinucleotide frequencies within each library. A similar analysis performed on Bacterial and Eukaryotic genomes sampled at random yielded entropies of 2.63 and 2.66 respectively.

The two samples produced more than 70 Mbp of sequence data from over 700,000 sequences, and there was no significant skew in the sequence data (as measured by dinucleotide frequency) when the data generated by pyrosequencing was compared to complete genome sequences.

### 16S rDNA analysis of the samples

**Figure 2:**
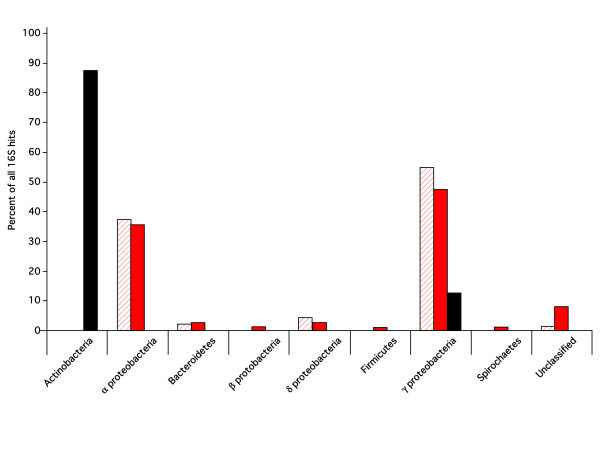
**Composition of the 16S rDNA sequences from the two samples and comparison of 16S sequences from the 454 libraries and a traditional clone library**. The percentage of all sequences from each library in each of the orders is shown for the 454-sequenced Black sample (solid black bars; n = 24), the 454 sequenced red sample (solid red bars; n = 76), and the PCR amplified clone library (hatched red bars; n = 91).

The two sequence libraries were compared to the 16S rDNA database from the Ribosomal Database Project[16]. As shown in Figure 2, the Black sample was dominated by Actinomycetales such as *Brevibacterium *and *Corynebacterium *that volatilize sulfur via an organic intermediate and can also break down complex heterocyclic and polycyclic ring structures[17,18]. In contrast, members of the Chromatiales, including the genera *Chromatiaceae*, *Thiobacillus*, and *Halothiobacillus*, dominate the Red sample. These chemoautotrophic Bacteria often use the Calvin-Benson-Bassham cycle to fix CO_2 _through the oxidation of iron or sulfur, and consequently they would be expected to be present in samples from an iron-rich deposit. These two communities are fundamentally different both from each other and from the community identified in the Iron Mountain metagenome[7]. The community in the Red sample has a much higher species richness than the Black sample, and the differences between the Soudan and Iron mines reflect the iron composition (hematite versus pyrite), temperature, and pH of the various environments[7].

A16S clone library was created from the Red sample to validate the 454 sequencing approach. Ninety-six clones were sequenced using traditional techniques, and compared to the 16S rDNA database from the Ribosomal Database Project [16]. The congruity between the 16S genes sequenced in the 454 library and the 16S sequences from the clone library, as shown in Fig. 2, is quite remarkable.

We also used the 16S sequences to evaluate the randomness of the library. An analysis of 160 bacterial genome sequences in the SEED database [15,19] with annotated 16S genes showed that about 1 in 10^5 ^bases is from a 16S gene. Based on this estimate, as a rule of thumb the Soudan samples are expected to contain approximately 3,000 bases of 16S sequence in total, or approximately 30 sequences. Twenty four sequences were found to have significant similarity (with an E value less than 1 × 10^-5 ^and a match of 50 bp or more) to 16S rDNA from the Black sample and seventy six sequences were found to have significant similarity to 16S rDNA from the Red sample.

### Metabolic potential from the metagenome library

**Figure 3:**
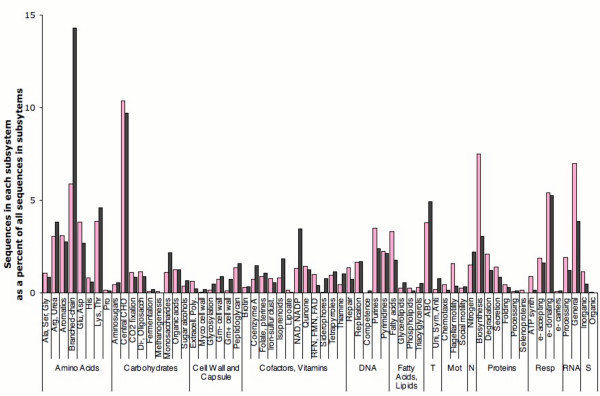
**Subsystems in the Red and Black Samples**. The occurrence of classes of subsystems is shown as a percent of all subsystems in each sample for the Red and Black samples. Notes and abbreviations: The subsystem class "Glu, Asp" also contains Gln and Asn. The subsystem class "Lys, Thr" also contains Met and Cys. CHO: Carbohydrates; sacch: saccharides; Extracell. Poly: Extracellular polysaccharides; Myco: Mycobacterial cell wall; Gm: Gram stain positive (+) or negative (-); Clust: clusters; RFN: Riboflavin; T: Transporters; Mot: Motility; N: Nitrogen; Resp: Respiration; e-: electron; S: Sulfur.

Sequences from both libraries were compared to the SEED database, a curated database of microbial genomes [15,19]. The annotations using the SEED interface primarily occur through the development of subsystems, a technique pioneered by the Fellowship for Interpretation of Genomes[15,20]. Subsystems are groups of genes that function together, such as the genes whose products are involved in a metabolic pathway, or the group of genes whose products make a cellular structure. A summary of the subsystem hits are shown in Figure 3, and all matches to subsystems are provided as supplemental data [see Additional files 1, 2 and 3]. These subsystems show that the pyrosequencing generates sequences that represent a large swathe of central metabolism in each of the environments. Common metabolic potential that is expected to be present in sulfur-utilizing chemoautotrophs is represented in the mine libraries, including the Calvin-Benson cycle, inorganic sulfur assimilation, amino acid biosynthetic genes, and so on. The comparison of the subsystem similarities suggested the simple hypothesis that groups of genes (or subsystems) important to a particular environment will be enriched in that environment. To distinguish between ecologically important differences and differences caused by sampling error, a method was devised to identify those subsystems that are statistically significantly overrepresented in one sample when compared to another [11].

### Subsystems enriched in the Black or Red samples

Table 2 shows subsystems that were determined to be statistically more common, with 95% confidence, in either the Red or Black samples from the Soudan mine. The subsystems that are overrepresented in a metagenome can yield significant insights into the microbial ecology of the environment. A few specific examples are detailed below.

**Table 2:** Subsystems statistically more likely to be present in either the Red or Black samples. These subsystems are more frequently found among sequences from either the Red or Black samples with a sample size of 5,000 proteins, 20,000 repeated samples, and *P *< 0.05.

***Red Sample (Oxidized, pH4.37, E_h_-8)***	***Black Sample (Reduced, pH 6.70, E_h_-142)***
**Amino Acids and Derivatives**	
Arginine biosynthesis	Urea decomposition
Tryptophan synthesis	Chorismate synthesis
Asp-Glu-tRNA(Asn-Gln) transamidation	Branched-chain amino acid biosynthesis
Histidine biosynthesis	Isoleucine degradation
	Leucine biosynthesis
	Leucine degradation and HMG-CoA metabolism
	Valine degradation
	Methionine salvage

**Carbohydrates**	
	Glyoxylate synthesis

**Cell Division and Cell Cycle**	
Cytoskeleton	

**Cell Wall and Capsule**	
	N-linked glycosylation in Bacteria
	Teichoic acid biosynthesis

**Cofactors, Vitamins, Prosthetic Groups, Pigments**	
Folate biosynthesis	Coenzyme A biosynthesis in pathogens
Methylglyoxal metabolism	Molybdopterin biosynthesis
Pyruvate metabolism I: anaplerotic rx, PEP	Carotenoids
Ubiquinone biosynthesis	Polyisoprenoid biosynthesis
Ubiquinone menaquinone-cytochrome c reductase	NAD and NADP cofactor biosynthesis global
Riboflavin metabolism	Coenzyme PQQ synthesis
	Pyrroloquinoline quinone biosynthesis
	Siderophore enterobactin biosynthesis
	Siderophore enterobactin biosynthesis and ferric enterobactin transport
	Thiamin biosynthesis

**DNA metabolism**	
DNA repair, bacterial	

**Fatty Acids and Lipids**	
Fatty acid metabolism	Glycerolipid and glycerphospholipid metabolism
Fatty acid oxidation pathway	

**Membrane Transport**	
ABC transporter maltose	ABC transporter ferrichrome
	ABC transporter heme
	CbiQO-type ABC transporter systems
	Sodium hydrogen antiporter

**Metabolism of aromatic compounds**	
Phenylacetate pathway of aromatic compound degradation	Homogentisate pathway of aromatic compound degradation

**Motility and Chemotaxis**	
Bacterial chemotaxis	
Flagellum	

**Nitrogen Metabolism**	
	Denitrification

**Nucleosides and Nucleotides**	
De novo purine biosynthesis	
Ribonucleotide reduction	

**Protein Metabolism**	
Ribosome LSU bacterial	Phenylpropionate degradation
Ribosome SSU bacterial	
Translation factors bacterial	
Universal GTPases	
Protein degradation	

**Respiration**	
F0F1-type ATP synthase	NiFe hydrogenase maturation
Terminal cytochrome C oxidases	
Hydrogenases	
Membrane-bound Ni, Fe-hydrogenase	
Na(+)-translocating NADH-quinone oxidoreductase and rnf-like group of electron transport complexes	
Respiratory complex I	
Respiratory dehydrogenases 1	

**RNA metabolism**	
Polyadenylation bacterial	
RNA polymerase bacterial	
tRNA aminoacylation	

**Stress response**	
Glutathione redox metabolism	
ppGpp biosynthesis	

**Sulfur Metabolism**	
Sulfate assimilation	

**Virulence**	
Resistance to fluoroquinolones	

**Figure 5:**
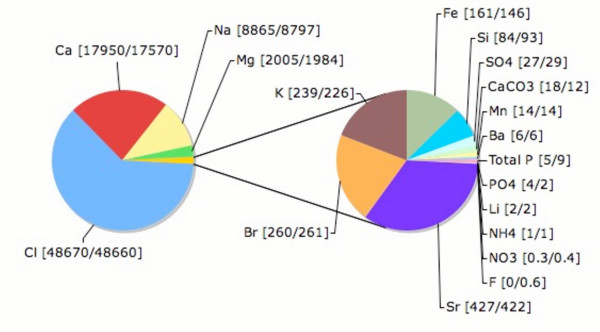
**Cations and Anions found in the Soudan Mine**. The pie chart shows the abundance of cations and anions found in the mine. The numbers in parentheses are the concentrations (in ppm) of each ion in the "Black" and "Red" samples respectively. The minor ions are shown expanded in the rightmost pie.

Several subsystems involved in iron uptake and utilization such as siderophores and ABC transporters for ferrichrome and heme are more common in the Black sample. The overall concentration of iron at the two sites was similar (Table 3; Figure 5). However, the iron in the Black sample is present as either Fe^2+ ^dissolved in the water or as ferroan [(Mg,Fe)_6_(Si,Al)_4_O_10_(OH)_8_]. In either case, the ferrous iron can not be assimilated biologically, and the microbes are forced to scavenge for the limited ferric iron (Fe^3+^) available. In contrast, in the Red sample, goethite [FeO(OH)] is present and ferric iron is more readily available for biological utilization. The Black sample is enriched for amino acid degradation pathways and microbes may be assimilating nitrogen or carbon through these pathways. It is not currently apparent from where free amino acids would be supplied.

**Table 3:** Water chemistry from Soudan Mine. No significant differences were found for Ca, Mg, Na, K, Li, Al, Mn, Sr, Ba, Si, Cr, Co, Ni, Cu, Zn, As, Se, Rb, Cd, Cs, Pb, total alkalitity, lactate, acetate, formate, chlorate, oxalate, and trace elements.

	***Black***	***Red***
Temp (°C)	10.9	10.9
pH	6.70	4.37
redox (mV)	-142	-8
Fe (ppm)	161.5	146.3
Total N (ppm)	1.510	1.280
• NH_4_	1.22	0.91
• NO_3_	0.29	0.36
• NO_2_	<0.10	<0.10
SO_4 _(ppm)	27.4	29.4
PO_4 _(ppm)	4.1	1.8
B (ppm)	186	70
Mo (ppm)	2.59	0.68
W (ppm)	3.82	0.91
Tl (ppm)	1.90	0.52
U (ppm)	1.01	0.20

**Figure 6:**
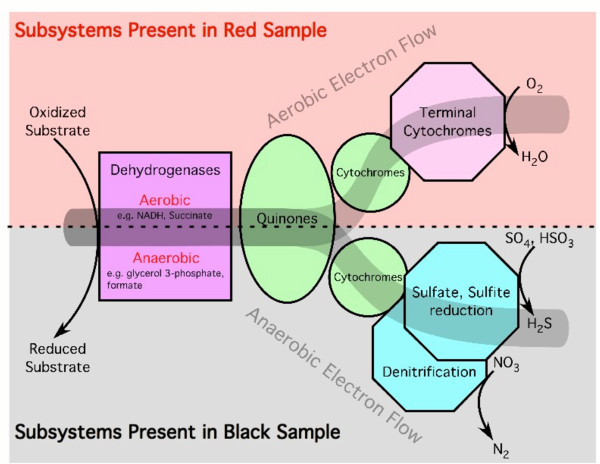
**Respiration in aerobic and anaerobic environments**. Among other potential pathways in the Soudan mine, electrons are transferred from hydrogenases to either cytochromes and then to oxygen to produce water in an oxidative environment, or via nitrate and nitrite reductases (denitrification) in anaerobic environments. Genes encoding the hydrogenases, respiratory complexes, and terminal cytochromes of the aerobic sample were significantly more abundant in the Red (oxidized) sample, while genes encoding the hydrogenases and denitrification genes were more abundant in the Black (reduced) sample. After Vassieva, O. [25]

The respiratory complexes and cytochrome-C oxidases are more commonly found in the sample from the oxidized environment (the Red sample; Table 2). Respiration proceeds via multiple electron transfer steps (Figure 6). In an aerobic environment, electrons are passed from hydrogenases to quinones (e.g., ubiquinone, quinone, menaquinone, and plastoquinone) and then to cytochromes resulting in the conversion of oxygen to water. In anaerobic environments the electrons are shuffled through nitrite and nitrate reductases, reducing NO_3 _first to NO_2 _and then to N_2 _gas. The Black sample is enriched for these denitrification genes suggesting that the latter pathway predominates while the Red sample is enriched for components of the aerobic respiratory pathway. Moreover, the Black sample had a lower concentration of free nitrate than the Red sample, presumably because nitrate is being used as an electron acceptor during respiration (although nitrite was below the level of detection in both samples; Table 3).

This analysis demonstrates that by combining pyrosequencing, subsystems analysis, and comparative metagenomics the microbiology of different environments can be correlated with the chemistry and hydrogeology of those environments to identify significant ecological differences between them.

### Comparisons between Soudan and Iron Mountain communities

A previous study used Sanger sequencing to determine the metagenome of the Iron Mountain community[7]. The environmental differences (such as the difference in temperature) account for the predominant differences between the microbial communities. The organismal differences are reflected in the individual biochemistries of the samples [see Additional files 4 and 5]. For example, the AMD metagenome contains significantly more occurrences of Archaea-specific subsystems such as those involved in protein biosynthesis than the Soudan samples. The AMD sample has a preference for CO_2 _fixation and simple carbohydrate metabolism when compared to either of the Soudan samples. There are also many currently unexplained differences between subsystems found in these environments that must relate the biology of the organisms to the chemistry of the environment.

### Comparisons between Soudan and other metagenome sequences

**Figure 4:**
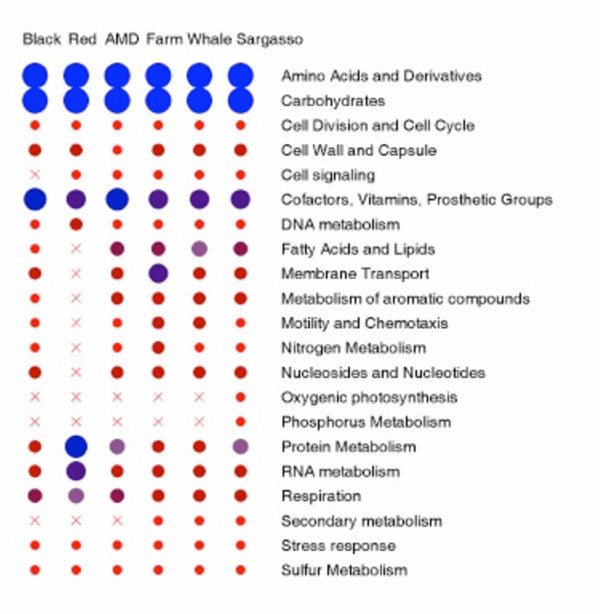
**Subsystems present in different metagenome sequences**. The subsystems present in the Soudan samples, the Iron Mountain AMD sample, the Minnesota Farm and the Sargasso Sea are shown grouped by family. The red x corresponds to very low abundance or complete absence of that family of subsystems. The size of the circle represents the proportion of sequences seen within that family of subsystems.

The SEED database used for these studies contained 351 subsystems. The vast majority (83%) of subsystems were present in one or more of the sequenced metagenomes, and over half (52%) of the subsystems are present in every metagenome. A comparison of the subsystem classification reveals trends between the metagenomes (Figure 4). For example, oxygenic photosynthesis is prevalent in samples that are naturally illuminated such as the Sargasso Sea[10]. This analysis also suggested that phosphorous metabolism is more prevalent in oceanic surfaces rather than terrestrial environments. Comparisons of the Minnesota Farm metagenome[6] with the Soudan Mine metagenomes, also from Minnesota, showed important differences in the production and consumption of secondary metabolites, membrane transport, and fatty acid metabolism. The complete lists of statistically significantly different subsystems between both Red or Black samples and each of the previously published metagenomes are supplied as supplemental material [see Additional files 4 and 5].

## Conclusion

This is the first metagenome analysis performed using pyrosequencing, which is approximately 10 to 30 times cheaper than current Sanger sequencing. Pyrosequencing also eliminates the need for cloning, thus removing the potential for both aberrant recombinants in the surrogate host and for cloning-related artifacts such as counterselection against potentially toxic genes such as those found on phages[2]. The main concerns with current pyrosequencing technology are the short length of sequence fragments (average of 105 bp in this study), and the requirement to use whole genome amplification to generate sufficient DNA for sequencing from environmental libraries The former may make it difficult to accurately assemble genomes in the absence of a scaffold, while the later may bias these analyses. Our preliminary unpublished data suggests that the whole genome amplification bias is minimal, and is preferentially towards the ends of linear pieces of DNA (Haynes, Rayhawk, Edwards, Rohwer; unpublished). Since these biases are applied equally to both libraries, they will be negated during the comparative study to highlight differences between metagenomes. Nonetheless, the short fragments are sufficient to determine statistically significant differences between metagenomes that reflect the most likely biology occurring in each environment. The low cost, high yield of pyrosequencing combined with statistical analyses on the abundance of subsystems in the samples allows the rapid identification of key processes driving the metabolism of different environments.

The systems approach of integrating biology, chemistry, and geology has yielded significant insights into the metabolism of two environments in the Soudan Mine. The oxidized sample is using aerobic respiratory pathways while the reduced sample is using anaerobic pathways. Nitrogen assimilation, iron acquisition, and sulfur metabolism are all differentiated between these two samples from close proximity within the same mine. However, many more significant differences between the samples remain unexplained by our current knowledge of bacterial physiology and metabolism. Explaining these differences will be a grand challenge for the future. By combining pyrosequencing, subsystems analysis, comparative metagenomics, and statistics, Occam has used his razor on metagenomics.

## Methods

### Sample collections, microbial enumeration, and DNA extraction

Samples were collected from several sites in the Soudan Mine. This analysis concerns the sample collection at two sites on Level 27 (714 m below the surface; Figure 1). Water and sediments were sampled from the two locations shown in Figure 1 giving the "Black" (reduced) sample and "Red" (oxidized) sample. Microbes were concentrated from these samples by filtration with 0.22 μm Sterivex units. Microbial counts were enumerated by staining the samples with SYBR-Gold (Invitrogen, Carlsbad, CA) and visualization with an epifluorescent microscope [21]. DNA was extracted from the microbial sample using either the Ultra Clean Soil DNA Kit or Power Soil Kit (MolBio, Boulder, CO). The DNA was amplified with GenomiPhi (GE Healthcare, Piscataway, NJ) in an Eppendorf thermal cycler (Eppendorf, Westbury, NY) using multiple reactions containing 50–100 ng of the isolated DNA as template and the manufacturer's recommended protocols. After amplification, the resulting DNA was purified with silica columns (Qiagen, Valencia, CA) and concentrated by ethanol precipitation. The DNA was resuspended in water to a final concentration of 0.3 mg/ml. Approximately 10 μg of each sample was sequenced using the pyrosequencing technology (454 Life Sciences, Branford, CT).

Bacterial-specific primers 27F (5'-AGAGTTTGATCMTGGCTCAG-3') and the universal 1492R primer (5'-TACGGYTACCTTGTTACGACTT-3') [22] were used to amplify the 16S rDNA genes. PCR products were cloned into the pCR^®^4-TOPO^® ^vector as recommended by the manufacturer (Invitrogen, Carlsbad, CA).

### Water and mineral analyses

Water samples were collected by filtering the water through 0.2 μm filters into clean bottles. Field measurements of pH, E_h_, temperature and conductivity were conducted *in situ*. The sediment samples were collected as slurries with a pipette resting on and in the sediments. Those slurries were transferred to clean centrifuge tubes, allowed to settle by gravity and then the fluid was decanted.

Major anions in the water were determined by GC (Dionex IGS-2000, Sunnyvale, CA) and major and trace elements by ICP/MS (Thermo Electron PQ ExCell, Franklin, MA). The mineral identifications are based on XRD (Bruker-AX D500 X-ray Diffractometer, Germany) measurements. The X-ray peaks were relatively small. Much of the sediment was apparently not well crystallized.

### Sequence analysis

The unassembled sequences provided by 454 were compared to the SEED database using the BLASTX algorithm on the Teragrid cluster at Argonne National Laboratories[15,23]. All BLAST searches were performed using an expect value cutoff of 1 × 10^-5^. At this cutoff approximately 3 of the observed hits would be expected to occur at random[23].

The BLASTN algorithm was used to identify 16S genes from release 9 of the RDP database [16,24]. These BLAST searches were also performed using an expect value cutoff of 1 × 10^-5 ^and a minimum sequence match length of 50 nt.

### Statistical analyses of metagenome datasets

The statistical analysis of subsystems present in each sample was performed essentially as described elsewhere [11]. The presence or absence of subsystems between two data sets was determined using 20,000 replicates of samples of 5,000 subsystems each. The 95% confidence interval for the median was constructed using the 0.025 and 0.975 percentiles.

## Authors' contributions

LW, MB, and FR collected the samples from the mine. DP, MS, SA, and CA performed chemical and hydrogeological analyses. MH extracted the DNA and processed samples for sequencing, BR-B, and RE performed computational and statistical analysis. RE authored the manuscript, and all authors edited and commented on the paper.

## Supplementary Material

Additional File 6 A gzip compressed archive of the fasta files (those ending .fa.gz) and quality scores (those ending .qual.gz) of sequences from the Red samples as supplied by 454, Inc. Click here for file

Additional File 7 A gzip compressed archive of the fasta files (those ending .fa.gz) and quality scores (those ending .qual.gz) of sequences from the Black samples as supplied by 454, Inc. Click here for file

Additional File 1 **Table1SRed**. Two lists (one for the Red Sample and one for the Black Sample) describing all the similarities found in the data. The table has the following columns: "Classification I" and "Classification II" are hierarchical classifications of the subsystems. "Subsystem" is the name of the subsystem [25]. "Functional Role" is the role of the protein in the subsystem to which the sequence from the Soudan Mine was similar. "Occurrence" is the number of times that a functional role is found in each sample. The text files have the data as tab separated items, and the file ending .xls has the same data in Microsoft Excel format. Click here for file

Additional File 2 **Table1SBlack**. Two lists (one for the Red Sample and one for the Black Sample) describing all the similarities found in the data. The table has the following columns: "Classification I" and "Classification II" are hierarchical classifications of the subsystems. "Subsystem" is the name of the subsystem [25]. "Functional Role" is the role of the protein in the subsystem to which the sequence from the Soudan Mine was similar. "Occurrence" is the number of times that a functional role is found in each sample. The text files have the data as tab separated items, and the file ending .xls has the same data in Microsoft Excel format. Click here for file

Additional File 3 **Table1S**. Two lists (one for the Red Sample and one for the Black Sample) describing all the similarities found in the data. The table has the following columns: "Classification I" and "Classification II" are hierarchical classifications of the subsystems. "Subsystem" is the name of the subsystem [25]. "Functional Role" is the role of the protein in the subsystem to which the sequence from the Soudan Mine was similar. "Occurrence" is the number of times that a functional role is found in each sample. The text files have the data as tab separated items, and the file ending .xls has the same data in Microsoft Excel format. Click here for file

Additional File 4 **Table2S**. The occurrence of subsystems in either the Red Sample or the Black Sample were compared to the subsystems found in the following metagenomes: AMD [7], Farm [6], Whale (all three whale falls combined)[6], the SEED non-redundant database [11], and the Sargasso Sea [10]. For each pair wise comparison the subsystems that are more likely to be found (*P *> 0.95) in either of the samples are shown, along with the sample that the subsystem is more likely to be found in. Subsystem names and classification are as found at [25]. The text file has the data as tab separated items, and the file ending .xls has the same data in Microsoft Excel format. Click here for file

Additional File 5 **Table2S**. The occurrence of subsystems in either the Red Sample or the Black Sample were compared to the subsystems found in the following metagenomes: AMD [7], Farm [6], Whale (all three whale falls combined)[6], the SEED non-redundant database [11], and the Sargasso Sea [10]. For each pair wise comparison the subsystems that are more likely to be found (*P *> 0.95) in either of the samples are shown, along with the sample that the subsystem is more likely to be found in. Subsystem names and classification are as found at [25]. The text file has the data as tab separated items, and the file ending .xls has the same data in Microsoft Excel format. Click here for file
